# Li^+^ Insertion in Nanostructured TiO_2_ for Energy Storage

**DOI:** 10.3390/ma13010021

**Published:** 2019-12-19

**Authors:** Mara Serrapede, Umberto Savino, Micaela Castellino, Julia Amici, Silvia Bodoardo, Elena Tresso, Angelica Chiodoni

**Affiliations:** 1 Center for Sustainable Future Technologies - Istituto Italiano di Tecnologia, via Livorno, 60-10144 Torino, Italy; umberto.savino@iit.it (U.S.); elena.tresso@polito.it (E.T.); 2 Department of Applied Science and Technology (DISAT)-Politecnico di Torino, C.so Duca degli Abruzzi, 24-10129 Torino, Italy; micaela.castellino@polito.it (M.C.); julia.amici@polito.it (J.A.); silvia.bodoardo@polito.it (S.B.)

**Keywords:** nanostructured materials, titanium dioxide, energy storage, Li-ion anode, Li-ion batteries

## Abstract

Nanostructured materials possess unique physical-chemical characteristics and have attracted much attention, among others, in the field of energy conversion and storage devices, for the possibility to exploit both their bulk and surface properties, enabling enhanced electron and ion transport, fast diffusion of electrolytes, and consequently high efficiency in the electrochemical processes. In particular, titanium dioxide received great attention, both in the form of amorphous or crystalline material for these applications, due to the large variety of nanostructures in which it can be obtained. In this paper, a comparison of the performance of titanium dioxide prepared through the oxidation of Ti foils in hydrogen peroxide is reported. In particular, two thermal treatments have been compared. One, at 150 °C in Ar, which serves to remove the residual hydrogen peroxide, and the second, at 450 °C in air. The material, after the treatment at 150 °C, results to be not stoichiometric and amorphous, while the treatment at 450 °C provide TiO_2_ in the anatase form. It turns out that not-stoichiometric TiO_2_ results to be a highly stable material, being a promising candidate for applications as high power Li-ion batteries, while the anatase TiO_2_ shows lower cyclability, but it is still promising for energy-storage devices.

## 1. Introduction

In the past 20 years great efforts have been made toward the study, growth and development of nanostructured materials for electrochemical energy storage, because they have the peculiarity to access both surface and bulk properties, thus enhancing the storage performances. The use of nanostructured materials is especially reported for Li-ion batteries [1,2,3,4] in which the short path lengths for electronic and ionic transport generate excellent cycling stability and fast charge/discharge rates. Moreover, nanostructured materials are of great interest for those devices, which represent an intermediate state between Li-ion batteries and supercapacitors, because they usually combine both Li-ion pseudo-capacitance and bulk-intercalation. In this view, nano-structuration allows us to tailor power and energy density as a function of degree of crystallinity, phase, particle size, morphology, and porosity.

Regarding TiO_2_-based materials, particular interest is devoted to understanding the relationship between the degree of crystallinity and the electrochemical performance as negative electrodes in energy storage.

Ivanov et al. [5] attributed the higher Li-ion diffusion rate in amorphous TiO_2,_ with respect to the crystalline one, to the larger amount of disorder and defects, which provide bigger channels for the ion transport. The crystalline electrodes usually are more rigid and undergo significant mechanical stress, which negatively affects the stability over time [6] and inhibits large amount of intercalated ions [7]. Fang et al. [8] observed for crystalline and amorphous TiO_2_, obtained with the same morphological features, that a higher rate capability is reached in the latter one.

Here we present a detailed voltammetric analysis of lithium insertion in defective and stoichiometric titanium dioxide, with the aim to compare and quantify the charging mechanisms of defective TiO_2_ with respect to stoichiometric anatase. Remarkably, our TiO_2_ samples were obtained by simple thermal oxidation of Ti foil in a 111 vol hydrogen peroxide solution (30% (w/w) in H_2_O) at low temperature. This method allows us to prepare highly porous metal-oxide electrodes avoiding the inclusion of conductive components and binding agents. We adopted the method developed by Dunn et al. [9] by separating the overall stored charge into pseudo-capacitive fast processes and diffusion-controlled ion-insertion in the bulk. Highly defective TiO_2_ and anatase show different behaviors: the first exhibits good cycling performance that is very promising for application in systems that require high stability, while the capacity values obtained with anatase confirm its applicability in energy storage devices.

## 2. Materials and Methods

### 2.1. Material Synthesis

TiO_2_ samples were prepared by thermal oxidation of commercial titanium foils (99.7% purity, Sigma-Aldrich, Milano, Italy, 0.25 mm thickness). Prior to the synthesis, surface contaminations have been removed from the Ti plates by ultrasonic bath in acetone (99% purity, Sigma-Aldrich), and, then, in ethanol (99% purity, Sigma-Aldrich). The oxidation process occurred in a 20 mL closed vial filled with hydrogen peroxide (30 wt %, Sigma-Aldrich) in which the Ti foil was put in vertical position. The temperature was set at 80 °C and kept constant for 24 h. After that, samples were extracted from the vial and gently rinsed with deionized water. The resulting material is a self-standing electrode composed by a Ti bulk with a surface-oxide nanostructure, a few microns thick. The complete removal of interstitial peroxide and water was obtained by heating the samples at 150 °C for 2 h in Ar atmosphere (T150). Well-organized, crystalline, white anatase TiO_2_ was prepared by thermal treating the as-oxidized foils in air at 450 °C for 1 h (T450).

### 2.2. Material Characterization

The morphological characterization was performed with a field-emission scanning electron microscope (FESEM, Supra 40, ZEISS, Oberkochen, Germany). Transmission electron microscope (TEM) Tecnai F20ST (ThermoFisher Scientific Instruments (former FEI), Hillsboro, Oregon, OR, USA), equipped with a field emission gun (FEG) and high-angle annular dark field (HAADF) detector, operating at 200 kV, was employed for the lower scale analysis of the samples. For these measurements, the samples were scratched, dispersed in an ethanol solution and then drop coated onto a standard holey carbon coated Cu grid.

The chemical composition was unraveled through X-ray photoelectron spectroscopy (XPS) by using a PHI 5000 VersaProbe system (Physical Electronics, Inc. (PHI), Chanhassen, MN, USA). Monochromatic Al K_α_ (1486.6 eV) was used as X-ray source, and C 1s peak (284.5 eV) was used as reference for the calibration.

The surface area was calculated measuring nitrogen adsorption–desorption isotherms on a Quadrasorb SI (Quantachrome Instruments, Anton Paar Quantatech, FL, USA) in liquid nitrogen, and treating the results using the Brunauer−Emmet−Teller (BET) method.

### 2.3. Electrochemical Characterization

The electrochemical characterizations were carried out in a three electrode cell (PAT-cell supplied from EL-cell, Hamburg, Germany) in deaerated 1 M LiPF6 in EC:DMC with a Li foil and a Li ring as counter and reference electrodes, respectively. The samples have been dried at 100 °C overnight to remove any trace of water before any measurement. The electric contact of the sample with the cell cage was made by scratching its back, thus exposing the titanium substrate, and placing it above the lower stainless steel plunger of the PAT-cell. The presented experiments were performed with a potentiostat/galvanostat (PGSTAT302N from Metrohm, Utrecht, The Netherlands) using Nova 2.1 software for the acquisitions. Cyclic voltammetry was performed from the open circuit potential (VOC) of the cell first catholically, in order to reduce the electrode and to store charge through the insertion of Li^+^. Then the sweep was reversed re-establishing the valence of TiO_2_ and discharging the cell by the extraction the ions. Cyclic voltammograms were performed at various scan rates from 50 µ·Vs^−1^ to 3000 μ·Vs^−1^ between 0.5 and 3 V vs. Li/Li^+^. The cycling stability of the device, on the contrary, was tested through galvanostatic charge and discharge in coin cells on Arbin instrument. Each coin-cell was cycled at 1 h (1C) five times and then at 12 min (5C) ninety-five times. After 150 repetitions, the experiment was stopped. The currents applied in the galvanostatic experiments were estimated from the cyclic voltammograms: they were calculated as the average currents recorded during the anodic sweep of the cyclic voltammograms, in which the sweep took 1 h (1C) and 720 s (5C). In Figure 6b, “raw data” were those directly measured from the galvanostat and they were labeled with small dots. The capacity values, labeled with large dots, instead, were obtained by averaging the capacities in each repetition loop for each rate (average of five cycles at 1C as crossed points and average of ninety-five cycles at 5C as empty points).

## 3. Results and Discussion

**Figure 1:**
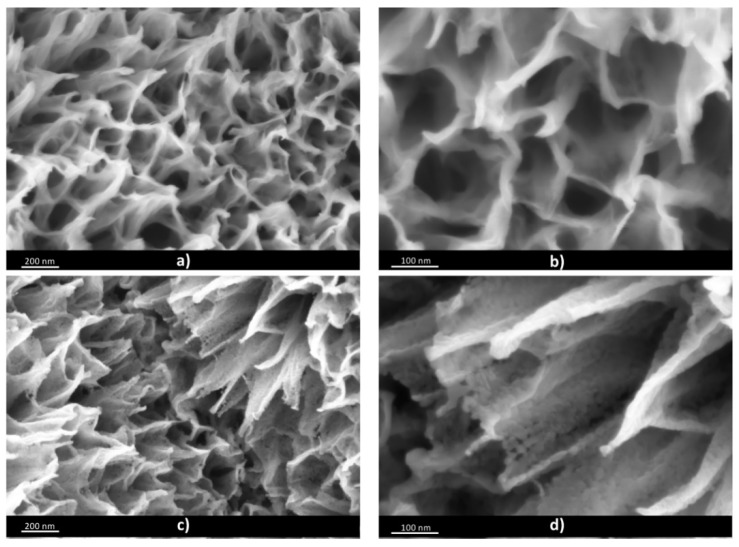
Field emission scanning electron microscopy (FESEM) characterization at two different magnifications of (**a**,**b**) T150 and (**c**,**d**) T450.

In Figure 1, the field emission scanning electron microscopy (FESEM) characterization of the samples T150 and T450 was reported. In Figure 1a,b, the material composed by flakes, few nanometers thick, arranged in a porous 3D structure, is shown with two different magnifications. The observed material porosity results from the gas evolution during the oxidation process in hydrogen peroxide.

In Figure 1c,d the FESEM characterization for sample T450 was reported at the same two magnifications. It is possible to observe a material with the same 3D arrangement as T150, but with the flakes composed by small crystals, interconnected along the flake wall. These crystals were the result of the calcination step operated in air at 450 °C.

**Figure 2:**
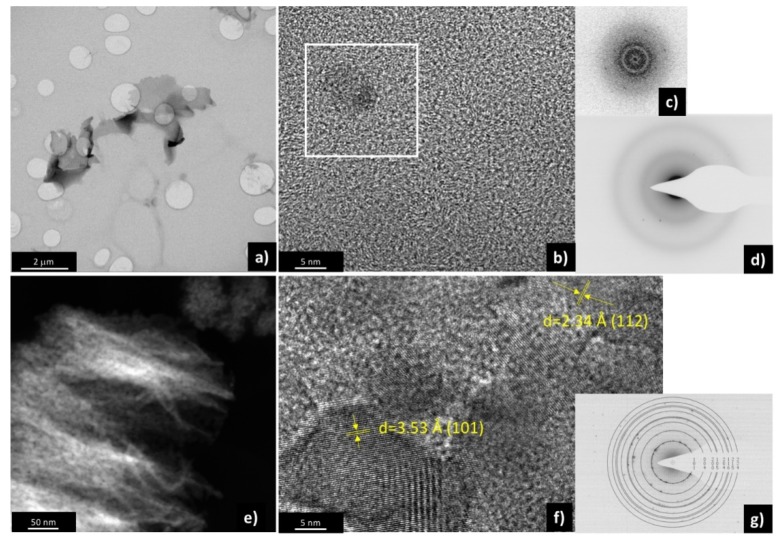
(**a**) Bright-field scanning transmission electron microscopy (STEM), (**b**) high resolution TEM (HRTEM), (**c**) fast Fourier transform (FFT) of the squared white region, and (**d**) electron diffraction pattern of sample T150. (**e**) high-angle annular dark field (HAADF)-STEM, (**f**) HRTEM, and (**g**) electron diffraction pattern of sample T450.

To shed more light on the structural properties of the two materials, scanning transmission electron microscopy (STEM), high resolution TEM (HRTEM) and electron diffraction (ED) were performed. Figure 2a shows the STEM-bright field image of a representative flake of sample T150, well transparent to the electrons, and quite homogeneous in thickness. HRTEM was performed on different flakes, to better understand the structural properties of such material. It resulted in being amorphous, with some very small (around 5 nm or less) crystalline domains present in the amorphous matrix, compatible with anatase crystalline structure. This was evidenced by the fast Fourier transform (FFT) in Figure 2c, related to the white squared region in Figure 2b, containing a small crystal, in which the dots represent the evidence of the crystalline orientation of the particle, and by the electron diffraction pattern of some flakes reported in Figure 2d, constituted by rings (the amorphous part) and by dots (the crystalline part).

Figure 2e shows a HAADF-STEM image of sample T450; it represents a nanostructured material, composed by small crystals organized in flakes, as was already put in evidence from FESEM characterization. HRTEM in Figure 2f shows crystals in the range 10–30 nm, identified as TiO_2_ anatase. This is also confirmed by the electron diffraction pattern shown in Figure 2g.

**Figure 3:**
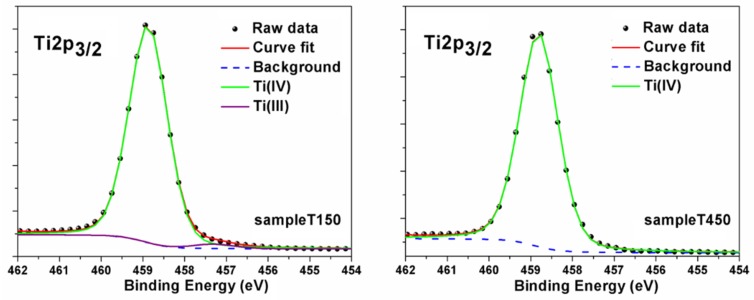
Ti 2p_3/2_ X-ray photoelectron spectroscopy (XPS) peaks for T150 (**left**) and T450 (**right**) samples. Curve fitting results have been reported, by showing raw data (scatter black dots), curve fit (red line), Shirley background function (blue dash line), and Ti components (green line for Ti(IV) and purple line for Ti(III)).

XPS analysis was carried out in order to study the surface chemical properties of both samples. From survey spectra (not reported) it was evident the presence of Ti, O, and C; the latter element was due to contamination related to ambient exposure from samples production to analysis session. We focused our attention on HR spectra for all the elements. Here we reported only Ti 2p signal (see Figure 3), since it is the one that gave information regarding Ti average oxidation states. The Ti 2p_3/2_ XPS core line spectrum for sample T150 (Figure 3—left panel), was almost identical to that expected for a Ti(IV) chemical shift [10], with a prominent peak located at 458.8 eV. However, by comparing and overlapping T150 and T450 experimental curves, a small shoulder was detectable only on the lower binding energy (BE) side of the signal spectrum for T150 sample, at 457.3 eV, which was the position usually related to Ti(III) oxidation state [11], while this bump was not present in the T450 one. In order to evaluate the relative percentage due to the Ti(III) component, we fixed the deconvolution parameters by setting the FWHM of the Ti(IV) component equals to 1.1 eV and the Gaussian-Lorentzian ratio to 80:20 for both samples. For sample T450, only one component was enough to reproduce the experimental data, while for T150 one, an extra component was necessary to perfectly overlap the raw signal. This second component represents 2% of the entire signal. So, we could state that while for sample T450 we were able to assign only a Ti(IV) component to the Ti 2p signal, as expected for pure anatase phase, sample T150 surface was not completely uniform, from the structural and chemical point of view, since we detected two different phases: 98% Ti(IV) and 2% Ti(III).

**Figure 4:**
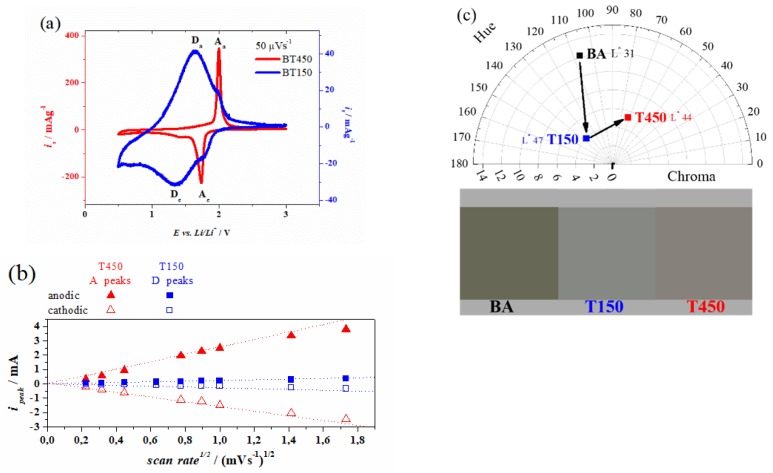
(**a**) Cyclic voltammograms of samples T150 in blue and T450 in red at 50 µV·s^−1^. A peaks refer to anatase phase and D peaks to the amorphous matrix. (**b**) Cathodic and anodic peak currents plotted against the square root of the scan rate. (**c**) CIE L*Ch color space by using D65 as illuminant, a standard observer at 10° and the CIE64 convention with the reproduced color of the samples as prepared (RT) and after each annealing temperature.

Electrochemical properties of the two samples were studied by cyclic voltammetry of Li-insertion. In the T450 sample, the presence of strong peaks (called A peaks) at 2.00 V and 1.74 V, ascribable to the anatase phase, confirmed the phase purity of this sample. The voltammogram of T150 instead, exhibited two pair of peaks: A and D. Even though the structural properties of this sample are quite complex, it is possible to confirm the presence of small crystallites of anatase, rising the two small A peaks, in a matrix that produces stronger D peaks, as already evidenced in the TEM analyses. Those anodic and cathodic peaks that have their maximum intensities at 1.65 V and 1.35 V respectively show a large peak separation, as shown in Figure 4, generated from an irreversible electrochemical reaction. The Li-insertion and extraction at those potentials produced extremely wide peaks, due to the absence of preferential channels for the intercalation that in turn provided different energy sites. These peaks represent a voltammetric signature that usually is observed in amorphous electrodes [12,13,14]. Remarkably, the reduction of T150 covers a potential range wider than fully crystalline T450, and the sample delivered a substantial amount of charge at a potential below 1.7 V, suggesting a higher Li molar fraction with respect to that of pure anatase (x = 0.5) [15,16,17].

The formal potential of D peaks, i.e., 1.50 V, is extremely close to the formal potentials of black TiO_2_ (B) that are 1.52 V and 1.59 V [18,19], but the measurements recorded by cyclic voltammetry suggest a different nature: at rates lower than 1000 µV·s^−1^, TiO_2_ (B) usually exhibited two pair of peaks, S1 and S2, which were mainly capacitive. The D peaks in T150 instead were unique even at 50 µV·s^−1^ and the charging and discharging processes appeared mainly diffusive. Not all the authors identify a capacitive charging in TiO_2_ (B), in fact Mason [20] observed a fully diffusion-controlled trend while Dylla [21] and Laskova [22] suggest a mixed capacitive and diffusion-controlled charging.

In order to address the complex behavior of the Li intercalation in the electrodes, the method of Dunn [9] was employed: the peak current is analyzed at a fixed potential and obeys to:

i(V) = *k_a_* ν + *k_b_* ν^1/2^ (1)

where *k_a_*ν is the capacitive current contribution associated to the storage of Li^+^ at the surface and to the bulk pseudo-capacitance, and *k_b_* ν**^1/2^** is the diffusion controlled current, which is attributed to Li^+^ insertion in the crystalline structure. It is clear from Figure 4b, that a linear relationship was present only when the peak currents at the anodic and cathodic potentials (mentioned above for both the samples) were proportional to the square root of the scan rates. This indicates in the anode a prevalent diffusion-controlled charging and discharging through Li^+^ in the crystalline lattice. Therefore, the diffusion coefficient was estimated by using the equation for an irreversible electrochemical reaction at a planar electrode:

*i_p_* = 0.4958 *n* F *A C D*^1/2^ ((α n_a_ F)/RT) ^1/2^ ν ^1/2^, (2)

where *i_p_* is the peak current in amperes, *n* and *n_a_* are taken as unity, *A* is the BET inner surface area in cm^2^, *C* is the maximum concentration of Ti reduced from 4+ to 3+ in the lattice, in which x = 0.5 for the anatase phase; and *D* is the chemical diffusion coefficient for Li^+^ ions in cm^2^/*s.*

*α*, the transfer coefficient, is estimated by applying the equation for irreversible waves:

α = (1.857 RT)/(*n_a_* F|*E_p_* − *E_p/2_*)|), (3)

where *n_a_* is the number of electrons involved in the rate determining step, *E_p_* is the peak potential, and *E_p/2_* is the half-peak potential. The other symbols have their conventional meanings.

For the diffusion coefficient calculation, we also assumed that the thickness of reduced Ti^3+^ states is sufficiently thin with respect to the particles so that the particle surface can be considered planar.

From the slopes of Figure 4b calculated by linear fitting and applying Equation (1), the diffusion coefficients have been estimated to be about 2 × 10^−17^ cm^2^/s in Li^+^ extraction for T150 and 8 × 10^−16^ cm^2^/s and 3 × 10^−16^ cm^2^/s in extraction and insertion respectively, for T450, all at 1000 µV·s^−1^. The diffusion coefficient in insertion of T150 was not estimated because of the impossibility to calculate the transfer coefficient in the reduction wave.

It is possible to notice that for both T150 and T450 samples, the Li^+^ proceeds faster in extraction with respect to the insertion, as demonstrated by the slopes in Figure 4b.

A better comprehension of the physical and electrochemical properties exhibited by the T150 and T450 samples can be obtained by examining the colorimetric properties. In fact, the oxidation of titanium foils in hydrogen peroxide produces samples, which—before annealing treatments—are extremely dark in color. In Figure 4c these samples are indicated as “BA”, they clearly evidence the colorimetric behavior of the black titania (TiO_2_ (B)). The BA samples have not been examined in this work since, for applications in electrodes of electrochemical storage devices, the peroxide molecules have to be removed by annealing treatments. After the annealing at 150 °C, the samples became lighter (ΔL* = +16), the yellowish disappears (Δh = +30), and the samples became duller (ΔC = −8), showing a deep grey color. After the second annealing at 450 °C, the lightness and chroma remained almost stable, but a massive change in hue was observed (Δh = −78). The variation of the interaction between the illuminant D65 and the samples at different annealing temperature were quantified and shown in the CIE L*Ch color space in Figure 4c.

**Figure 5:**
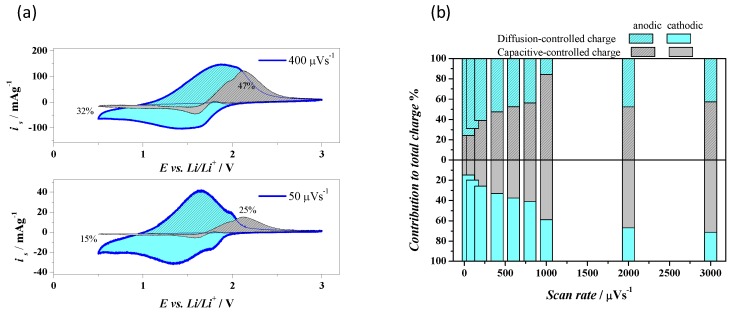
(**a**) Cyclic voltammograms of sample T150. The voltammograms are displayed for scan rates 400 µV·s^−1^ (upper) and 50 µV·s^−1^ (lower). The thick blue lines correspond to experimentally obtained voltammetric currents. The grey areas and the cyan areas represent calculated capacitive and diffusion-controlled contributions, respectively. The patterned areas represent the anodic charge and plane areas the cathodic charge. (**b**) Percentage of the capacitive and diffusion-controlled processes to the overall charges at all scan rates recorded.

Due to the unique nature of T150 sample, in which an amorphous matrix contains small crystallites of anatase, the Dunn’s method was employed to distinguish the nature of the charging currents at each potential of the voltammograms. According to the method, herein we reconstructed the voltammograms (Figure 5a) for the capacitive contribution (grey area) with respect to the raw voltammetric data of the overall currents (blue thick line). Therefore, the cyan area contribution reconstructed the diffusion-controlled charges.

The voltammograms at 50 and 400 µV·s^−1^ are shown in Figure 5a: it is clear that the capacitive contribution was remarkable only during the discharge of the electrode, confirming a larger diffusion constant during the extraction of Li^+^. Figure 5b also shows the percentage of the capacitive and diffusion-controlled processes to the overall charges collected at all scan rates recorded, either for the cathodic or anodic sweeps (the latters are patterned). The calculated contributions of capacitive and diffusion-controlled percentages with the extracted (Q^+^) and inserted (Q^−^) charges are reported in Table 1. The pseudo-capacitive contribution in T150 dominated only for scan rates higher than 600 µV·s^−1^, but at this scan rate the extracted charges were only half with respect to those measured at 50 µV·s^−1^.

During the reduction of Ti^4+^ to Ti^3+^, the pseudo-capacitive contribution dominated only at scan rates higher than 1000 µV·s^−1^ with a stored charge of only 30% with respect to that at 50 µV·s^−1^. The reasons of this inequality during the insertion and extraction of Li^+^ in this modified TiO_2_ relied on the fact that the tetragonal structure of TiO_2_ (poor in Li), containing distorted TiO_6_ octahedra [23], was much more favored and stable with respect to the orthorhombic structure (rich in Li); therefore the oxidation results tended to be faster and at lower overpotentials.

The partial capacitive nature of T150 could be either attributed to the presence of the anatase crystallites, which are nano-sized: for TiO_2_ below 10 nm in size, it is well known that the capacitive contributions become relevant [24], as shown in Figure 2b. This approach was not employed to analyze the behavior at different scan rates of the sample T450 because the insertion and extraction of ions were fully diffusion-limited at the solid state, since the crystallites were larger than 10 nm, as previously observed in TEM.

**Figure 6:**
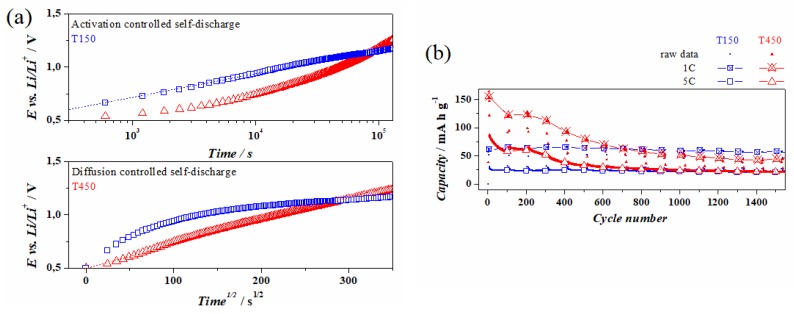
(**a**) Self-discharge profiles of samples T150 and T450 in logarithmic (upper) and square root (lower) time scale. (**b**) Cyclability tests at 1C and 5C.

To better investigate the potentialities/peculiarities of these materials, the self-discharge of T150 and T450 in a device-configuration was investigated. They were placed at their maximum charging potential (0.5 V) for 24 h and then let discharge at open circuit potential (OCP) by recording their drop in potential during the self-discharge. It is clear from Figure 6a that the potential in T450 decayed linearly with respect to the square root of time, indicating a phenomenon in which the mass transport plays the crucial role and the diffusion from the bulk solution to the electrode leads the anode to discharge [25]. This suggests the presence of some impurities at low concentration in the electrolyte, which are electro-active in the potential range 0.5 ÷ 2.8 V vs. Li/Li^+^, and that shuttle the electrons from the working electrode to the counter electrode in which reduction occurs. Differently, in T150 the main process, which leads to the loss of charges at OCP, resulted in being under activation control. This evidence comes out from the linearity of the potential decay with respect to the Log of the time [26].

It is well known that in amorphous materials, the cation mole fraction is higher with respect to the crystalline counterpart and this could generate a large concentration of highly reactive species leading to a faster discharge. These results should be taken into account when designing a full cell. In fact, the faster self-discharge of this electrode as anode could lead to a sudden return from the maximum potential of the full cell to 0 V.

To assess the stability of the two materials, aging cycling was performed. From the galvanostatic cycling of T450 in Figure 6b it is possible to observe that in the first five cycles at 1C, the theoretical capacity of TiO_2_ anatase (168 mAh·g^−1^ [27,28]) was almost reached (155 mAh·g^−1^) and that it decreased to become stable up to 200 cycles at a value of 125 mAh·g^−1^. From 300 to 1200 cycles, the capacity decreased monotonically and then it stabilized for cycles up to 1300 with a capacity of 47 mAh·g^−1^. The same trend was observed when cycled at 5C, where it started from 65 mAh·g^−1^ and stabilized at the 700th cycle with a capacity of 25 mAh·g^−1^. In T150 instead, the capacities were much less, being at 1C 65 mAh·g^−1^ and at 5C 25 mAh·g^−1^, but no fading was observed.

For both samples, it was observed that up to 1550 cycles, the coulombic efficiencies were much higher than 80% (Figure 6b).

## 4. Conclusions

In this paper we presented an extensive study of the electrochemical properties of TiO_2_-based electrodes for energy-storage applications. The materials were prepared via a simple and scalable technique, i.e., by thermally oxidizing a titanium foil in hydrogen peroxide. This synthetic path led to the formation of a porous three-dimensional structure formed by few nanometer thick flakes, as proved by FESEM imaging.

Two different thermal treatments were carried out aiming at obtaining (i) a defective structure, by annealing the as-grown material in Ar atmosphere at 150 °C, or (ii) stoichiometric TiO_2_, by air annealing at 450 °C. The former structure resulted to be constituted by anatase nanocrystals embedded in an amorphous matrix, rich in Ti-reduced states corresponding to Ti (III). On the other hand, the latter was found to be formed by defect-free anatase crystals, with a mean size of tens of nanometers.

The Li-ion intercalation was investigated; as a result, the defective material exhibited a higher Li molar fraction with respect to the crystalline one, with a prevalent diffusion-controlled charging-discharging, mediated by Li^+^.

A faster extraction, with respect to insertion, of Li-ions was observed in both samples.

For this reason, the Dunn’s method was used to unravel the Li-ion intercalation processes, thus allowing us to reconstruct and separate the capacitive and diffusive contributions of the overall charges, giving a comparative quantification among them.

As a final result, it turned out that while the anatase sample successfully reached the theoretical capacity of TiO_2_, keeping it stable for 200 cycles, the defective material exhibited a lower capacity, however succeeding in maintaining it constant even after 1550 cycles. This characteristic makes this material promising in application requiring a higher stability, such as high power Li-ion batteries.

## Figures and Tables

**Table 1:** Comparison of capacitive and diffusion-controlled charge storage contribution to the total stored charge for sample T150. The overall total anodic and cathodic charges are reported for samples T150 and T450 at different scan rates.

	T150						T150	
	Cathodic			Anodic			Cathodic	Anodic
	Q^−^	Diffusion-controlled	Capacitive-controlled	Q^+^	Diffusion-controlled	Capacitive-controlled	Q^−^	Q^+^
µVs^−1^	C·g^−1^	%	%	C g^−1^	%	%	C∙g^−1^	C∙g^−1^
**50**	**722**	**85**	**15**	**530**	**76**	**24**	945	913
100	492	80	20	447	69	31	843	850
200	389	74	26	374	61	39	804	795
**400**	**314**	**67**	**33**	**305**	**52**	**48**	–	–
600	275	62	38	269	47	53	709	702
800	246	59	41	243	44	56	693	684
1000	221	41	59	217	16	84	669	660
2000	168	33	67	167	48	52	586	578
3000	141	29	71	140	43	57	536	527
